# Carcinome épidermoïde vulvaire: pourquoi surveiller un lichen scléro-atrophique

**DOI:** 10.11604/pamj.2015.21.48.6018

**Published:** 2015-05-22

**Authors:** Inssaf Ramli, Badredine Hassam

**Affiliations:** 1 Service de Dermatologie et Vénérologie, CHU Ibn Sina, Université Mohammed V, Rabat, Maroc

**Keywords:** Ulcération, carcinome épidermoïde, la vulve, lichen scléro-atrophique, Ulceration, squamous cell carcinoma, vulva, atrophic lichen sclerotiorum

## Image en medicine

Le carcinome épidermoïde (CE) de la vulve représente 3 à 5% des cancers génitaux de la femme. Il peut survenir sur des lésions de dysplasies liées à une infection à papillomavirus ou sur des lésions de lichen scléreux. Cliniquement, il s'agit d'une ulcération douloureuse ou prurigineuse suintante reposant sur un fond érythroplasique siégeant au niveau de la face interne des grandes lèvres dans 40% des cas. Le caractère superficiel de l'ulcération peut être responsable d'un retard de consultation et de prise en charge. L'histologie permet la confirmation diagnostique en objectivant des kératinocytes atypiques franchissant la membrane basale, regroupés en lobules centrés par des globes cornés. Le CE de la vulve est de mauvais pronostic avec un envahissement essentiellement ganglionnaire et locorégionale. Quant à la prise en charge thérapeutique optimale, il n'y a pas de consensus. La chirurgie reste la pierre angulaire du traitement. La radiothérapie externe et la curiethérapie interstitielle gardent une place prépondérante dans l'arsenal thérapeutique. Nous rapportons le cas d'une femme de 34 ans, suivie depuis 5 ans pour un lichen scléro-atrophique à localisation vulvaire, qui consultait pour une tumeur ulcéro-bourgeonnante, bien limitée, de 3 cm de diamètre, à localisation vulvaire. L’étude histologique était en faveur d'un carcinome épidermoïde. Un bilan d'extension n'a pas révélé de localisation secondaire. La recherche d'une infection sexuellement transmissible était négative. Une vulvectomie totale avec curage ganglionnaire inguinal bilatéral ont permis la guérison de la patiente sans récidive ni de localisation secondaire avec recul de 18 mois.

**Figure 1:**
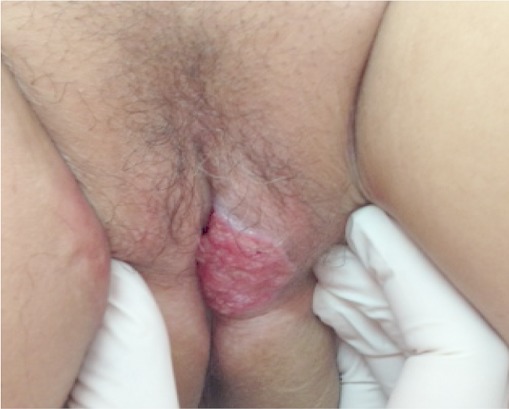
Tumeur ulcéro-bourgeonnante de la vulve

